# COMPLETE – a school-based intervention project to increase completion of upper secondary school in Norway: study protocol for a cluster randomized controlled trial

**DOI:** 10.1186/s12889-018-5241-z

**Published:** 2018-03-09

**Authors:** T. Larsen, H. B. Urke, I. Holsen, C. H. Anvik, T. Olsen, R. H. Waldahl, K. M. Antonsen, R. Johnson, M. Tobro, B. Brastad, T. B. Hansen

**Affiliations:** 1 Department of Health Promotion and Development, University of Bergen, Bergen, Norway; 2 Nordland Research Institute, Bodø, Norway; 3 Oxford Research, Kristiansand, Norway

**Keywords:** Randomized controlled trial, Drop out, Upper secondary school, Psychosocial learning environments

## Abstract

**Background:**

Drop out from upper secondary school represents a risk for the future health and wellbeing of young people. Strengthening of psychosocial aspects of the learning environment may be an effective strategy to promote completion of upper secondary school. This paper is a study protocol of a school based cluster randomized controlled trial (RCT) evaluating two school-based interventions, namely the Dream School Program (DSP) and the Mental Health Support Team (MHST). The interventions aim to improve psychosocial learning environments and subsequently school achievements and decrease drop-out and absence.

**Methods/Design:**

The COMPLETE RCT is aimed at youth in upper secondary school, grade 1 (age 15-16 years), and examines the effect of the combination of the DSP and the MHST; and the DSP only, compared with a comparison group on the following primary outcomes: student completion, presence, average grade, and self-reported mental health. Seventeen upper secondary schools from four counties in Norway were randomized to one of the three arms: 1) DSP and MHST; 2) DSP; and 3) comparison (offered DSP intervention in 2018/2019). The study will evaluate the interventions based on information from two cohorts of students (cohort 1 (C1) and cohort 2 (C2)). For C1, data was collected at baseline (August 2016), and at first follow-up seven months later. Second follow-up will be collected 19 months after baseline. For C2, data was collected at baseline (August 2017), and first and second follow-up will be collected similarly to that of C2 seven and 19 months respectively after baseline. Process evaluations based on focus groups, interviews and observation will be conducted twice (first completed spring 2017).

**Discussion:**

The COMPLETE trial is a large study that can provide useful knowledge about what interventions might effectively improve completion of upper secondary school. Its thorough process evaluation will provide critical information about barriers and points of improvement for optimizing intervention implementation. Findings can guide school development in the perspective of improving psychosocial learning environments and subsequent completion of upper secondary schooling.

**Trial registration:**

The trial was retrospectively registered in the ClinicalTrials.gov register on December 22.2017: NCT03382080.

## Background

The value of completing upper secondary schooling is reflected in associations with higher education [1, 2], lower risk of later economic hardship [3], better mental and physical health, and higher life satisfaction among others [4, 5]. Therefore, drop out from upper secondary school represents a risk for the future health and wellbeing of young people [1], as well as societal costs [6–8]. Therefore, it is an explicit aim of authorities in a range of countries, including Norway, to ensure that as many young people as possible complete upper secondary school [6].

The reasons behind any youth’s decision to drop out of upper secondary schooling can be several, intertwined, complex and often have origins in early childhood [9]. Poor mental health is one important cause of upper secondary school drop-out and absence [10–15]. Therefore, special attention to youths’ mental wellbeing and health is needed to promote completion of upper secondary school.

Although conditions outside the school setting interfere with a person’s decision to drop out of upper secondary school, the role and responsibility of schools in striving for each student’s well-being and motivation in school is ubiquitous. Strengthening of psychosocial aspects of the learning environment may be an effective strategy to promote school satisfaction and completion of upper secondary school. The learning environment is the work environment of young people, and a good and inclusive psychosocial learning environment promotes health, wellbeing and learning [16–18].

Poor academic achievement is found to be another strong predictor of school dropout [19]. To stimulate students’ confidence in their own competence and efficacy and thus empower them as learners therefore seems particularly relevant in the school context. A study by Finn and Rock [20] found that academic resilience was partially explained by the extent to which students are actively engaged in school. Engagement in learning activities and in the broader school environment was seen as important antecedents of school achievement. Unlike such characteristics as SES or ethnicity, engagement may be manipulated; that is, educators can encourage engagement behaviors to increase a student’s chances of completing school successfully.

According to Baumeister and Leary [21], one of the central tasks and goals of human life is to sustain a network of close relationships characterized by mutual caring and pleasant, supportive interactions. Enabling a good psychosocial environment in schools through participatory activities stimulates the development of such relationships. In addition, Vinokur and van Ryn [22] concluded that interpersonal conflicts that are expressed in undermining behaviors appear to have a strong concurrent impact on mental health. Building positive and caring relationships in school might prevent negative behavior.

One aspect of the psychosocial learning environment is the teacher-student relationship. A recent systematic review of the role of teacher-student relationships concludes that the teacher-student relationship can play an important part both as risk factor for poor mental health and as a protective factor for good mental health [23]. The review also concludes that positive teacher-student relationships may prevent drop-out and reduce the rate of intention to drop out [23].

Peer relationships and the feeling of connection to peers is another central aspect of the psychosocial learning environment of students. Settertobulte and Gaspar de Matos [24] found that being liked and accepted by peers is crucial to young people’s health development. Those who are not socially integrated are far more likely to exhibit difficulties with their emotional health. Depending on the character of its psychosocial learning environment, the school can be both a risk factor and a protective factor for student’s subjective physical and mental well-being.

Although research exists suggesting positive associations between aspects of the psychosocial school and learning environment and mental health and success in upper secondary school, knowledge about what type of systematic work should be put in place to strengthen these associations is lacking. There is currently a call for targeted interventions to reduce dropout, and robust evaluation designs to measure the effect of these [6]. A meta-analysis of experimental and quasi-experimental studies to reduce dropout from upper secondary school [25], found significant effects of a range of ‘programme types’, indicating the promising role of systematic interventions to come to grips with challenges of dropout. However, the interventions are not described in the meta-analysis which makes knowledge about what elements in these program types work, hard to access. Also, only studies from the US, UK and Canada were included. An important insight from the study is however, the conclusion that implementation quality may be of more importance than what type of programme the intervention is.

A more recent systematic review by Lillejord et al. [6] builds on the review by Wilson et al. [25], and 26 additional studies published between 2010 and 2015. Their analysis concludes that effective interventions are focused on the need of building strong relationships based on confidence between students and teachers, between peers, and between leadership, teachers and other significant actors involved in the work for and with students [6]. Other important factors for successful interventions are ensuring well-established collaborations between involved actors and levels, broad support for the programs, early interventions, and ensuring systematic planning, implementation and evaluation [6].

Despite knowledge about the importance of the psychosocial aspects of the learning environment, the review by Lillejord et al. [6] could not identify any experimental studies with explicit focus on the role of the psychosocial school environment in relation to dropout, and further states that this is a knowledge gap that needs to be filled. A further knowledge gap relates to experimental and quasi-experimental studies on interventions to reduce dropout in a broader array of national and cultural contexts, as the majority of such studies originates from the US and UK [6, 25].

As part of the efforts initiated by the Norwegian Ministry of Education [26] to reduce drop out in upper secondary schooling, a call for research evaluating promising interventions through randomized controlled trials was issued in the spring of 2015. This led to the development of the research project which is described in this study protocol: *COMPLETE – Good psychosocial environments improve the completion of upper secondary school* [27].

The COMPLETE study is an evaluation of the effectiveness of the interventions, and these will be evaluated in a cluster RCT.

### Aims

The aim of the COMPLETE study is to determine the effectiveness of the DPS and MHST among adolescents in upper secondary school with respect to the primary outcomes of completion/drop-out, presence/absence, school grades, and mental health.

Specifically, the COMPLETE study will

1. Evaluate whether the DSP alone
  - increases completion
  - increases presence
  - improves school achievements
  - improves mental health and wellbeing
2. Evaluate whether the DSP and the MHST combined
  - increase completion
  - increase presence
  - improve school achievements
  - improve mental health and wellbeing

The COMPLETE study will also evaluate the effect of the DSP and MHST combined and the DSP alone against secondary outcomes of school satisfaction and loneliness.

**Fig. 1:**
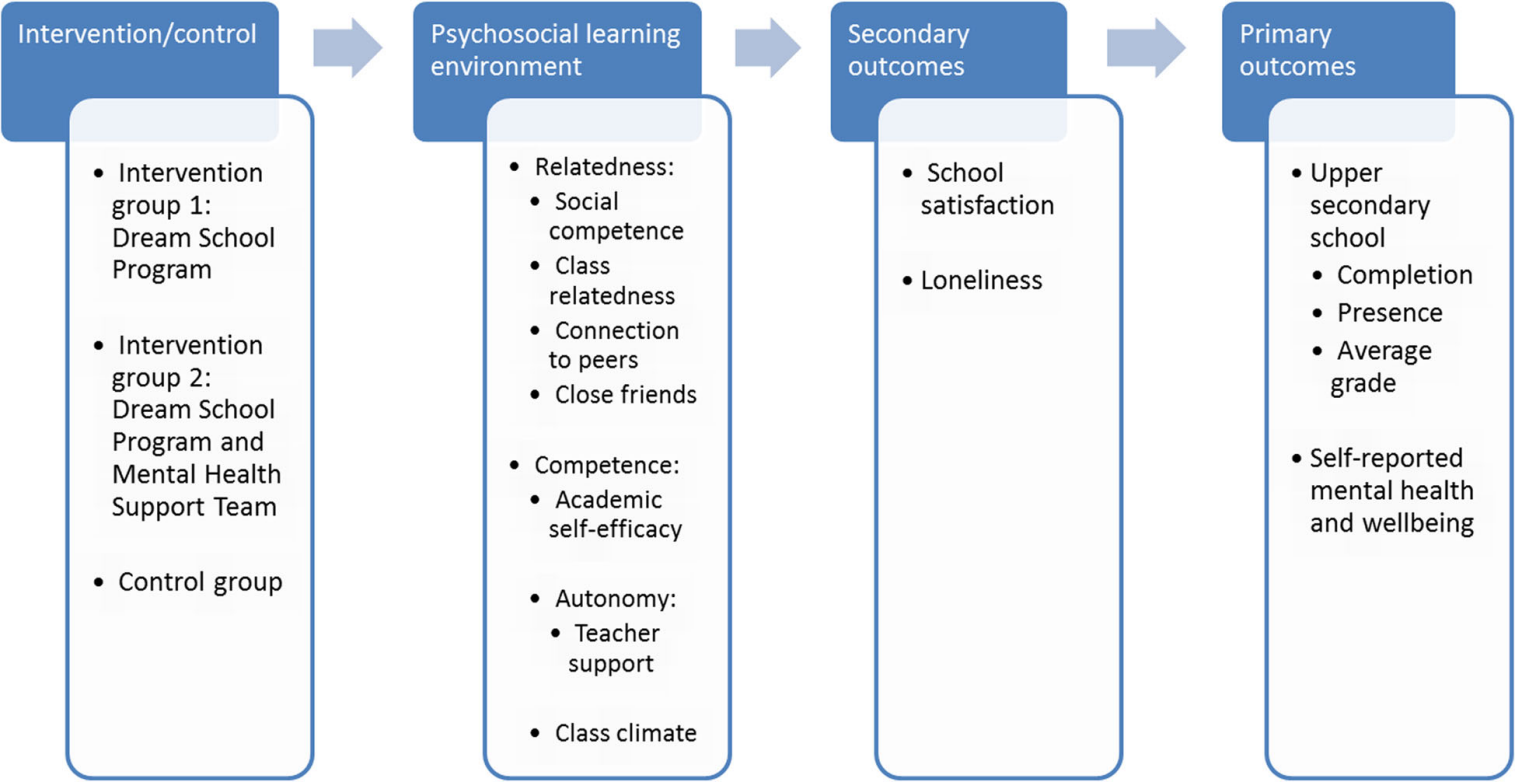
COMPLETE study program theory

Furthermore, according to the COMPLETE study program theory (Fig. 1), a range of mechanisms are in place to explain the potential effects of the interventions on the primary and secondary outcomes. These mechanisms are related to psychosocial aspects of the school and class environment, specifically to students’ experiences of relatedness, competence, autonomy, and class climate.

The aim of the present paper is to describe the study protocol of the COMPLETE RCT.

Finally, the COMPLETE study will through a process evaluation evaluate the implementation quality and fidelity of the DSP and MHST in the intervention schools.

## Methods

### Study design

COMPLETE is an ongoing school-based three-armed cluster RCT in upper secondary schools in four counties in Norway. The trial started in August 2016, and will end in June 2019. It follows two cohorts of students from when they start upper secondary school and until they graduate (C1) or until completion of second grade (C2). The study is non-blinded, and the design includes two intervention groups and a control group. Tables 1 and 2 outline the project data collection and intervention timeline.

**Table 1:**
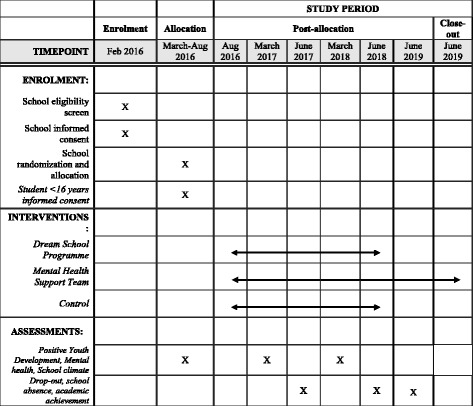
COMPLETE trial: Schedule of enrolment, interventions and assessments for Cohort 1 (C1)

**Table 2:**
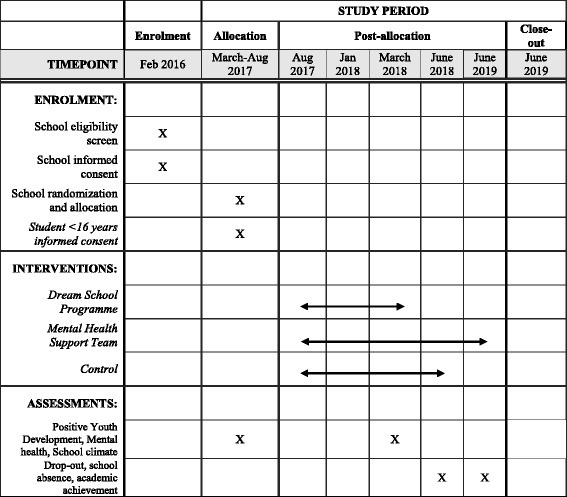
COMPLETE trial: Schedule of enrolment, interventions and assessments for Cohort 2 (C2)

### Sample

The size of the sample was based on two prerequisites. First, the implementation of the program in the intervention schools required training of several stakeholder groups. The NGO delivering the universal program set an upper limit to 12 secondary schools which would be manageable for them. Also, in further decisions on sample size, the power calculations with hierarchical structured data [28] followed the procedure applied in a prior comparable psychosocial intervention in Norwegian schools [29]: A hierarchical structure with students in class, a significance level of *p* ≤ .05, power of .80, classes with an average of 20 students, an assumed intraclass correlation of 0.05, and a minimum expected effect size of 0.25 on primary and secondary outcomes (expected effect sizes (Cohens *d*) in similar studies lies between .20 and .50, see meta analyses by Durlak et al. (2011). These power calculations indicated that a sample of 975 students and 49 classes was needed to detect a small effect size of .25.

### Recruitment of schools

Recruitment of schools started in November 2015 with information meetings and written invitations. All upper secondary schools in the four counties were invited to report their interest in participation in the trial. Eligibility criteria were not having been or currently being involved in the DSP or the MHST *or* in similar programs, *or* in other similar research projects. An invitation letter was sent to all schools in the counties via the county administration describing study aims, and project participation. The recruitment process lasted until February 2016. A total of 19 schools reported interest in joining the project, of which 17 schools met the eligibility criteria. These schools comprised approximately 3100 grade 1 students in total. All students are eligible for participation in the DSP and MHST, but for the effect evaluation and survey data collections, students with special needs and students in so called introductory programs (i.e. students with very limited or no knowledge of Norwegian language) were excluded. The final student N is based on students registered at each of the schools the final days before survey data collections. However, it should be noted that due to a high degree of natural fluctuation between schools and study programs the first week of each school year, it is expected that the study N from registries will deviate quite a bit from the survey sample. For C1 the N was 3003 grade 1 students and for C2 the N was 3022 grade 1 students. In C2, approximately 200 class teachers will also receive a survey.

**Fig. 2:**
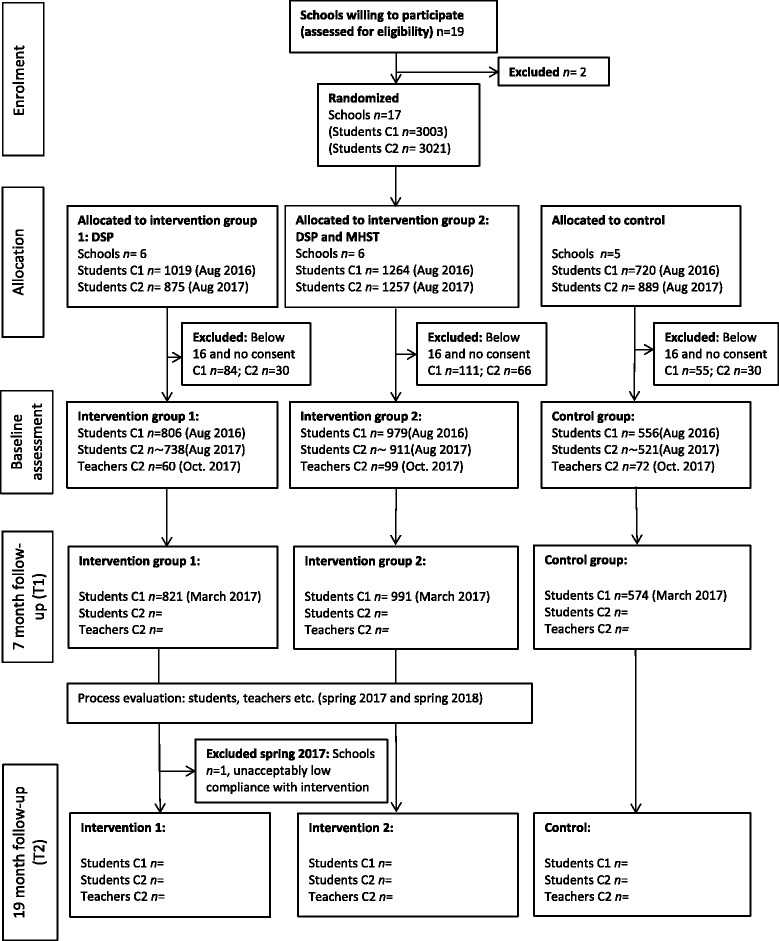
Flow chart of participation through the COMPLETE school based randomised controlled trial. N for baseline and follow-up assessments reflect the total number of students participating in each of the assessments, and not the number of participants participating in both assessments

Figure 2 presents the flow chart of participation through the COMPLETE trial per now.

### Randomization of schools

Representatives from Oxford Research who had no prior knowledge of the recruited schools allocated schools to the intervention or control groups according to a computer generated randomization list. To ensure an equal balance of intervention and control groups in each county, stratified randomization was practices in which schools were stratified by county. All schools were given a random number ranging from 0 to 1 by using a randomisation command in the statistics software of Stata 14. Stata generated a random number based on a chosen key. Next, the schools were sorted after the size of the random number in each stratum. The school with the highest number was appointed to the intervention group 1 – I1 (DSP only), the school with the second to highest number was appointed to intervention group 2 – I2 (DSP and MHTS), and the school with the third to highest number was appointed to the control group – C. This procedure was repeated until all schools in each stratum were appointed to a group.

### Participation

All first grade students in the DSP schools receive the intervention, as this is an integrated whole school program. Similarly, all students in the MHST schools have the opportunity to come in contact with the MHST if they need it as the MHST are part of the student services.

### Consent procedure

Informed active consent by parents is required for the participation of students below 16 years of age at time of survey data collection. The project administration only has access to student, and not parent contact information. Therefore written information about the study and survey was distributed to students via mail, email and sms with instructions to forward this to their parents. Consent could be given either by SMS to the project coordinator, or via an electronic response form in which parents could respond yes or no. For C1, a total of 820 students needed consent. The total response rate for consent request obtained for this group was 72%, and a total of 70% agreed that their child could participate. For C2, a total of 706 students needed consent. The total response rate for consent request obtained for this group was 81%, and a total of 80% agreed that their child could participate.

### Baseline assessment and follow up

Each student cohort will be assessed through survey on three (C1) or two (C2) occasions (Figs. 2 and 3):

1. Baseline: during the first or second week of school in the first semester of grade 1
  - C1: August 2016 (already completed)
  - C2: August 2017 (already completed)
2. First follow-up: in the second semester of grade 1
  - C1: March 2017 (already completed)
  - C2: March 2018
3. Second follow-up: in the second semester of grade 2
  - C1: March 2018

Registry data on drop-out, absence and school grades after grade 1, 2 and 3 will be linked to survey data. In addition, survey data from grade 1 teachers of the C2 will be collected in August 2017 and March 2018, to assess any change in teacher perceptions on their relational competence over the intervention period. However these data will be considered supplemental and not part of the quantitative effect evaluation.

### Intervention

Schools in the intervention groups (I1 and I2) started the planning of intervention implementation in the spring of 2016, and C1 students in the intervention schools received the interventions immediately after semester start in August 2016. The same procedure will be followed for the C2 students, starting upper secondary school in August 2017. Schools in the control group will be offered the DSP after completion of the second intervention year, starting in August 2018.

#### The Dream School Program (DSP)

The DSP is developed by the Norwegian NGO *Adults for Children* (AfC) [30]. The program is a whole school program, involving staff and students, with the aim of creating learning environments where students are confident and experience a sense of belonging, and where mental health is promoted [30]. A core aspect of the DSP is the training of peer leaders from grade 2 or 3, so called student mentors, as important executers of the program. The DSP involves specific core elements that must be carried out in order for it to be properly implemented. These are the Dream Class 1 and 2, and the Dream Class poster which involves making guidelines for a good psychosocial class environment. The student mentors are to be actively involved in collaboration with class teachers in carrying out these core elements. In addition, they welcome the new students on the first day of school, carry out theme gatherings, and are intended to be actively involved in creating meeting points for socialization. In addition, the school is encouraged to incorporate other activities that they usually carry out as part of a more systematic work for improving the psychosocial environment at the school. The effectiveness of the DSP has previously been evaluated in two pilot studies with promising effects on academic self-efficacy, teacher support, and intention to continue upper secondary school the following year [31, 32].

#### The Mental Health Support Team intervention (MHST)

The MHST intervention is developed in a collaboration between employees at Bodin upper secondary school in Norway [27] and researchers at Nordland Research Institute (NRI) [33].

MHST is also a whole school project but aimed at specific students at risk of dropping out. It is a systematisation of the student services through

1. Co-location of services and staff working in services
2. “One open door” to increase accessibility to the services and staff for students
3. Focus on the transition from lower to upper secondary school
4. Close follow-up of students at risk to ensure tailored help to each student
5. Early intervention and follow up when student starts being absent from school

This work demands cross- and multidisciplinary collaborations within the MHST, between MHST and school leadership, and between lower and upper secondary schools.

### Intervention delivery

#### DSP

The delivery of the DSP involved giving information to all DSP school leaders. Next, a resource group, comprised of five to seven representatives from the school leadership, student council and teachers were established and trained over two days each in workshops held by AfC. The resource groups in turn had responsibility for recruiting student mentors which were trained in student mentor gatherings by AfC staff. Student mentors were trained in workshops in the spring of 2016 for C1 and 2017 for C2. In May, June and August, of 2016 and 2017, workshops with school staff were carried out to prepare the schools for the school year with Dream school implementation. During the year, student mentor and resource group gatherings were organized separately for exchange of experience and inspiration between DSP schools. In addition, representatives from AfC visited a selection of the schools during the fall of 2016, and had phone conversations with all DSP schools in August 2016 and June 2017, in addition to email contact throughout the school year. They will follow the same procedure in the school year of 2017/2018.

#### MHST

The delivery of the MHST involved a different approach than the DSP as it is more of a structural intervention. Seminars with school leaders and team members were held in June and August 2016 to inform about the structure and core elements of MHST, and to discuss local solutions for implementation. Specifically, many schools had to undergo a reorganizing to ensure co-localization and “one open door”. Schools also vary in the employee situation with respect to number and type of workers associated with student services. Hence, establishing the team of 3-5 persons was one key priority in the early implementation phase. Schools with MHST have close follow-up with representatives from Bodin and with county coordinators (see more details on this under the section on Implementation quality and intervention fidelity below).

### Implementation quality and intervention fidelity

To ensure motivation and implementation quality, a range of dialogue meetings between project partners were held before and after recruitment of schools. Meetings between the project and county leaderships, and the intervention implementers (AfC and Bodin) were held before and after recruitment, and regular meetings are planned throughout the project period. Information meetings were held with school leadership teams, teachers and student representatives after recruitment.

To ensure intervention fidelity, yearly gatherings have been organised and will be organised throughout the project period separately for the peer mentors, the resource group members of each DSP school, and members of MHST to share experiences and motivate each other. In each gathering, each MHST is required to develop an action plan for their specific work the coming academic year. Similar gatherings are also organized at the county level with the county project coordinator involved. In the MHST gathering in June 2017, a representative from the leadership in each school with MHST was also present to secure understanding for the MHST work at the leadership level of the school. In addition, representatives from Bodin have phone calls with each team every other week to discuss progress, challenges and solutions.

### Measures

The primary outcomes in COMPLETE are increased completion of, and presence at upper secondary school, academic achievement, and mental health and well-being (Table 3). Data on the three former are obtained for each student participating in the study from county registries, and linked with survey data. More specifically, data on the completion of the first, second and third grade of upper secondary school; days and hours of time absent from school in the first, second and third grade of upper secondary school; and average grade obtained by each student after the first, second and third grade of upper secondary school will be collected from registries. To classify a student as dropout, the student must have quit school, and not just switched schools or study programs.

**Table 3:** Outcome measures

	Measure/definition	Source of measure	Data source
Primary outcomes			
Dropout/Completion	Drop-out is indicated if the student has a) not completed upper secondary school within five years after enrolment in upper secondary school, or b) if the student quit school and is without any regular activity during the course of the study period, i.e. grade 1-3 of upper secondary school. Completion of school year is indicated if the student has passed all subjects at the end of the current school year	Registry data	County registry data
Presence/Absence	Average days and average hours absent the past school year	Registry data	County registry data
Academic achievement	Average grade at closure of each school year in project period	Registry data	County registry data
Mental health	Short form of Symptom Check List (SCL-5)	Tambs et al., 1993 [34]	C1 and C2 Student survey
Mental wellbeing	Warwick-Edinburgh Mental Wellbeing Scale (WEMWBS)	Clarke et al., 2011 [35]	C2 Student survey
Secondary outcomes			
School satisfaction	Multidimensional Student’s Life Satisfaction Scale: Three items from the school satisfaction dimension	Huebner, 1994 [37]	C1 and C2 student survey
Loneliness	Loneliness Scale	Mittelmark et al., 2004 [39]; Kraft & Loeb, 1997 [38]	C1 and C2 student survey
Mediators			
Relatedness	Wichstrøm Social Competence Scale	Wichstrøm, 1995 [41]	C1 and C2 student survey
	Teacher and Classmate Support Scale	Torsheim et al., 2000 [44]	C1 and C2 student survey
	Positive Youth Development Connection Scale	Geldhof et al., 2014 [43]; Lerner et al., 2005	C1 and C2 student survey
	How many close female friends do you currently have?	Single item	C1 and C2 student survey
	How many close male friends do you currently have?	Single item	C1 and C2 student survey
Autonomy	Ten items from the Learning Climate Scale	Black & Deci, 2000 [45]	C1 and C2 student survey
Competence	How do you think you will perform academically this school year?	Single item	C1 and C2 student survey
	Academic Self-Efficacy Scale	Roeser et al., 1996 [46]	C1 and C2 student survey
Class climate	Caring Climate Scale (CCS)	Newton et al., 2007 [47]	C1 and C2 student survey

Data on mental health and wellbeing are obtained through surveys based on validated measures. Specifically mental health is measured by the short form of the Symptom Check List (SCL-5) [34], and mental wellbeing is measured by the Warwick-Edinburgh Mental Wellbeing Scale (WEMWBS), previously validated for use in adolescent populations in the UK [35], and in the Norwegian setting in an adult primary health care patient population [36].

Secondary outcomes in the trial include school satisfaction, measured through three items from the school dimension of the Multidimensional Students’ Life Satisfaction Scale [37] and loneliness [38–40] (Table 3). These data are also obtained for each student through surveys and validated measures.

Mediators in the trial include measures of psychosocial aspects, specifically measures of experienced relatedness [41–44], teacher autonomy support [45], competence [46], and climate [47] in the school, class and other contexts of the youth. These data are obtained for each student through surveys and validated measures. See Table 3 for details on the measures.

Moderators or background variables include student gender (male/female/other), ethnicity, socioeconomic status (student reported parental education level and family economic standing), and student line of schooling (generalised or vocational).

In addition, data on several other indicators relevant to youth social and emotional life are collected through the surveys. However, in this study protocol, only the most relevant indicators to the COMPLETE study aims are described.

### Data collection procedures

#### Effect evaluation

*Survey data* are obtained through electronic questionnaires developed in the survey program Enalyzer. Data are collected during class under administration of staff from Oxford Research [48]. Questionnaires are available in both Norwegian written languages (Norwegian bokmål and Norwegian nynorsk). One class hour (45 min) are allocated to completion of questionnaires, but as far as possible it is facilitated for spending more time for students who need it.

*Registry data* are obtained from county or school registries. The county project coordinators retrieve and handle the registry data to ensure anonymity of students through unique ID numbers and encryption codes. When anonymised, Oxford Research links the registry data with survey data.

#### Process evaluation

The process evaluation builds on two rounds of qualitative interview studies and school visits in 12 schools (the COMPLETE intervention schools) encompassing 19 subdivisions. The studies are conducted in the spring of 2017 (completed) and of 2018. They encompass qualitative interviews, both individual and group interviews, with school leadership (headmaster), subdivision leaders (e.g. study program leaders), class teachers, resource groups for the DSP, student mentors (DSP), students in the DSP, the MHST (counsellors, school nurses, team coordinators) and students in the target group for MHST. Every study (both 2017 and 2018) encompasses approximately 120 interviews and includes around 300 different persons. Every interview has a duration of approximately one hour. Every school visit covers three days and includes both interviews and observation of common areas like the canteen, social zones, outdoor areas, administration area and the teacher common room. Specific interview guides are developed for the different target groups in the study. These are structured interview guides with follow-up questions. The aim of the interviews is to capture the experiences the schools have with the implementation of the two interventions.

All data are stored according to guidelines and requirements for safe storage of data in the University of Bergen storage, SAFE.

### Statistical analyses

Findings will be reported according to the CONSORT guidelines for cluster RCTs [49].

Descriptive statistics from intervention and control groups will be presented comparing means, medians or percentage as appropriate for primary and secondary outcomes, potential mediators like relatedness, competence and autonomy, and potential moderators, like gender, family socioeconomic status, and ethnic background.

Effectiveness analyses will first be conducted according to the intention-to-treat principle, and adjusted for the clustered nature of the data. Further effectiveness analyses will be conducted stratified by implementation degree. Analyses of the primary and secondary outcomes will be conducted using latent growth models, specifically, linear mixed effects models and logistic mixed effects models as recommended [50, 51], and alpha levels will be set at *p* ≤ .05. The role of moderators and mediators will be assessed in latent growth model frameworks appropriate for these purposes. Potential differences in intervention effects between subgroups will be investigated in secondary analyses. Missing data will be handled with multiple imputations or FIML depending on what is appropriate in each analysis.

### Qualitative analyses

All interviews, both individual and group interviews, are audio-taped and transcribed verbatim. All transcripts are uploaded to the electronic software for qualitative data analysis, NVivo. All researchers involved in interviews and analysis have access to a common data file in NVivo. Here, the material is analysed thematically (through nodes). The analyses are structured according to the themes in the interview guides, the central elements in the interventions, as well as the implementation process for the interventions.

### Ethics, permission and consent

The study is approved by the Norwegian Centre for Research Data (NSD). Written (and oral) information about study aims and participation was and will be given to all participants prior to participation. Informed active consent by parents is required for the participation of students below 16 years of age at time of survey data collection, and the procedure for obtaining this is described above. Informed oral consent was practiced for the participation in interviews and focus groups.

### Data protection

Survey data are collected anonymously and the confidentiality of participants is assured by use of an encryption key for personal details, and this key is stored separately at the county level under the responsibility of the county project coordinator. Data collected in the project is securely stored in the University secure deposit for data storage, SAFE. Only the research partners from Oxford Research, University of Bergen and Nordland Research Institute have access to data, and access will be withdrawn from any researcher leaving the project before its completion.

The trial is funded by the Norwegian Ministry of Education.

### Dissemination

The project is required to deliver a midway report in December 2017 and a final report in January 2019 to the Ministry of Education. In addition, dissemination directly to partners in the participating counties and schools will be practiced. Researchers from University of Bergen, Nordland Research Institute and Oxford Research affiliated with COMPLETE will be mainly responsible for the writing of any publication from the project.

## Discussion

This paper aimed to describe the COMPLETE study, a cluster RCT including two intervention programs to improve youth mental health, completion of, presence at, and academic achievements in upper secondary school in Norway.

COMPLETE is innovative due to its three armed design, assessing the variation in effectiveness of implementing the entire prevention triangle including both universal and indicative and selective approaches versus a universal approach only, and comparing these approaches with a control group. Such a comprehensive effect evaluation is of value for education authorities with respect to where efforts should be intensified – is the universal approach enough, or are more systematic efforts recommended also for the at-risk groups of students?

### Trial status (01.11.2017)

The COMPLETE study is mid-way in its project period with three out of five student survey data collections completed, one out of two process evaluations completed, and one out of five linking of registry data on primary outcomes completed. The first report to the Norwegian Ministry of Education is due in January 2018, and the final project report is due in December 2019.

## Abbreviations

AfC: Adults for Children
C1: Cohort 1
C2: Cohort 2
DSP: Dream School Programme
MHST: Mental Health Support Team
RCT: Randomized controlled trial
SCL-5: Symptom Check List
WEMWBS: Warwick-Edinburgh Mental Wellbeing Scale
